# Medical Association of Central New York

**Published:** 1901-10

**Authors:** C. A. Van Der Beek

**Affiliations:** Rochester, N.Y., Secretary


### SOCIETY PROCEEDINGS.

### Medical Association of Central New York.

REPORTED BY C. A. VAN DER BEEK, M. D., Rochester, N. Y., Secretary.

THE thirty-fourth annual meeting of the Medical Association of Central New York, was held at the lecture room of the Buffalo Society of Natural Sciences, at the Library Building, Buffalo, N.Y., on the 27th and 28th days of August, 1901.

Tuesday, August 27th, the meeting was called to order at 10.30 a.m. by the president, Dr. LUCIEN HOWE, of Buffalo, N.Y.

The Secretary, Dr. C. A. VAN DER BEEK, upon being called to read the minutes of the last meeting, moved that they be omitted, as has been done heretofore, for the reason that they have been published in the Transactions.

Motion seconded. Carried.

The President then announced the standing committees.

*Business Committee.*—L. W. Rose, Rochester, chairman; S. F. Snow and A. F. Vadeboncoeur, Syracuse.

*Committee on Finance.*—William C. Krauss, chairman; William C. Phelps, Roswell Park, DeLancey Rochester, Stephen Y. Howell, Francis E. Fronczak, H. D. Ingraham, Charles G. Stockton, Eugene A. Smith, Eli H. Long, James W. Putnam, and Lucien Howe, all of Buffalo.

*Committee on Hotels.*—Floyd S. Crego, chairman; Alvin A. Hubbell and W. Scott Renner, Buffalo.

*Committee on Entertainment.*—William C. Krauss, chairman; Eli H. Long and William Warren Potter, Buffalo.

*Committee on Arrangements.*—Francis F. Fronczak, chairman; Henry D. Ingraham, Julius Ullman and Lorenzo Burrows, Buffalo.

*Committee on Credentials.*—Floyd S. Crego, chairman; F. F. Dow, Rochester, and W. A. Howe, Phelps.

*Publication Committee.*—C. A. Greenleaf, Wm. M. Brown, Rochester, and William C. Krauss, Buffalo.

The following members and delegates registered:

*Cayuga.*

| | | |
|---|---|---|
| Horley, J. P. | Palmer, John O. | Slocum, George |

*Chemung.*

| | | |
|---|---|---|
| Case, G. W. | Fisher, J. C. | Fulton, Cyrel |

*Chenango.*

Andrews, L. C.

*Cortland.*

| | |
|---|---|
| Higgins, F. W. | Kinyon, Benj. |

*Erie.*

| | | |
|---|---|---|
| Allen, Thos. G. | Grove, W. G. | McGuire, E. R. |
| Bartow, B. | Hauenstein, John | McGuire, Francis W. |
| Benedict, A. L. | Hinkel, F. W. | McMichael, Geo. H. |
| Brown, Geo. L. | Hopkins, H. R. | Mynter, Herman |
| Burrows, L., Jr., | Howe, Lucien | Park, Roswell |
| Cohen, B. | Howell, S. Y. | Phelps, W. C. |
| Colton, A. J. | Hubbell, A. A. | Pohlman, Julius |
| Congdon, C. E. | Ingraham, H. D. | Putnam, J. W. |
| Cott, Geo. F. | Jones, A. A. | Renner, W. Scott |
| Crego, Floyd S. | Johnson, Ray H. | Rochester,DeLancey |
| Dunham, Sydney A. | Keiser, Max | Stockton, C. G. |
| Fowler, Joseph | Krauss, Wm. C. | Ullman, Julius |
| Frederick, C. C. | Long, E. H. | Wall, Chas. A. |
| Fronczak, Francis E. | Lynde, U. C. | Wende, G. W. |
| Grove, B. H. | Mann, M. D. | York, G. W. |

*Kings.*

Hoag, David E.

*Livingston.*

Shanahan, W. T.

*Madison.*

Lum, W. T.

*Monroe.*

| | | |
|---|---|---|
| Allen, M. M. | Barron, W. M. | Brown, W. M. |
| Andrews, L. B. | Berkman, J. S. | Buck, Kathleen |

Conklin, Wm. L.

Dow, F. F.

Earl, Edgar H.

Elsner, Simon L.

Greenleaf, C. A.

Jones, William B.

Lane, Geo. A.

Maine, A. P.

Potter, E. B.

Potter, Marion C.

Rider, W.

Rockwell, C. S.

Sherman, James

Slayton, L. E.

Starr, Chas. S.

Sullivan, J. H.

Van der Beek, C. A.

Weigel, L. A.

*N. East, Pa.*

Putnam, B. H.

*Onondaga.*

Baun, Henry C.

Bryan, Geo. J.

Coon, C. E.

Craton, S. B.

Easton, F. E.

Gregg, Milton E.

Hadden, A. Whiting

Heffron, J. L.

Hotaling, A. S.

Kellogg, W. C.

Knowlton, F. P.

Larkin, S. E.

Law, W. F.

Lewis, G. G.

Loveland, B. C.

Murray, D. H.

Roberts, Charles S.

Sears, D. W.

Slingerland, I. N.

Slocum, F. W.

Snow, S. F.

Ten Eyck, P. Camp

Wallace, W. L.

Wynkoop, E. J.

*Ontario.*

Allen, D.

Beahan, A. L.

Clapper, Wm. B.

Jackson, C. O.

Howe, W. A.

Wheeler, S. R.

*Orleans.*

Bamber, R. W.

*Oswego.*

McKnight, E. E.
Rainier, E.

*Schuyler.*

Bennett, M. L.

*Toronto, Ont.*

Buck, Thos D.

*Waterproof, La.*

Andrews, D. Mark

*Wayne.*

Brandt, J. S.

Carmer, M. E.

Lapp, E. H.

Myers, J. F.

Putnam, J. W.

Veeder, M. A.

*Yates.*

Van Dyke, C. M.

The report of the treasurer, Dr. S. L. ELSNER, of Rochester was then read, and ordered printed. The report is as follows:

DR.

| | | |
|---|---|---|
| October 16, 1901. | To balance, cash on hand | $124.30 |
| | Receipts from dues | 165.00 |
| | Receipts from new members | 50.00 |
| | | $339.30 |

CR.

| | |
|---|---|
| By disbursements, October 16, 1900, to August 26, 1901 | $173.55 |
| By cash | 165.75 |
| | $339.30 |

The president's address being in order, the first vice-president, Dr. A. L. BEAHAN, of Canandaigua, was called to the chair.

The frequency of asthenopia in America was the title of the address, and was printed on page 117 of the September journal.

The following papers were then read and discussed:

A plea for the broader treatment of epilepsy, Wm. P. Spratling, Sonyea.

Salt starvation in the bromide treatment of epilepsy, A. Pierce Clark, Sonyea.

Rheumatism, L. A. Weigel, Rochester.

Hospitals in the smaller towns, A. L. Beahan, Canandaigua.

The use of anesthetics in children, by E. J. Wynkoop, Syracuse.

The science of medicine and the healing art, John L. Heffron, Syracuse.

During the reading of the papers, the circulation of blood through a bullock's heart was demonstrated by Dr. Knowlton, of Syracuse University, in an adjoining room.

The society adjourned at 1 o'clock p.m. for luncheon, tendered by the Buffalo physicians at the Tifft House.

In the evening, the Infant Incubator concession at the Pan-American Exposition tendered the association a special demonstration of their methods and life-saving inventions, which was much enjoyed and highly appreciated by all present. The members were then invited to a Mexican dinner at the "Streets of Mexico," and were there entertained by Wenona, the expert rifle shot of the Indian Congress. Appropriate remarks were made by several of the members, after which the members dispersed to enjoy the beauties of the exposition.

Second day, Wednesday, August 29, 1901.

The president called the meeting to order at 10.15 a.m.

At the request of the committee on entertainment, Mr. Frank C. Bostock, of the Trained Animal Concession, kindly consented to have Esau, the educated chimpanzee, appear at this session, and requested the members to make a careful comparative examination. Remarks were made by Drs. L. A. Weigel, Lucien Howe, W. A. Howe, Bernard Cohen, Wm. C. Krauss and others. A vote of thanks was tendered Mr. Bostock, Drs. Coney and Schenkein, of the Infant Incubators and to Mr. Cummins of the Indian Congress for courtesies shown the society.

The election of officers being in order, the society proceeded to ballot and elected the following: President, A. L. Beahan, Canandaigua; first-vice president, John L. Heffron, Syracuse; second vice-president, M. L. Bennett, Watkins; secretary, C. A. Greenleaf, Rochester; treasurer, Wm. M. Brown, Rochester.

The secretary read a communication asking the endorsement of this society for a physiological institution in Washington.

The President.—I have looked into this matter and am familiar with its endorsers. It has not seemed to me, at least, as a thing which we should sanction without some deliberation. If it is laid on the table for consideration another year, it strikes me that that would be a recognition of it, at the same time not committing ourselves. Is there any motion in regard to it in any way?

Dr. John L. Heffron, Syracuse—I move that the communication be received.

Motion seconded and carried.

Dr. Heffron.—I move that it be laid on the table for further consideration.

Motion seconded and carried.

Dr. Wm. C. Krauss, Buffalo.—At our last meeting an amendment to the Constitution was offered by Dr. E. B. Angell, of Monroe, chairman of the Committee on Revision of the Constitution, to the effect that hereafter we shall meet only in turn at Buffalo, Syracuse and Rochester. Dr. Angell being absent I therefore present that amendment to the Constitution at this time, and ask that it be voted upon. I move that hereafter this society shall meet annually in turn at Buffalo, Syracuse and Rochester, and at Auburn only on special occasions. Seconded. Carried.

Dr. JOHN L. HEFFRON.—The resolution which I now present I wish to have carefully considered by this body, and if it meets with approval, I should like to have it adopted:

Inasmuch as the present law regulating the practice of medicine in our state has been productive of great good to the people of the state by having developed a body of medical men, all of whom have shown proof that they are well grounded in the fundamental science of medicine, it is therefore desirable to correct such anomalies of the law as at present exist; therefore

*Resolved*, That the Medical Association of Central New York forward to the Committee on Legislation of the New York State Medical Society, through its secretary, the following memoranda for their careful consideration:

(1) By making the single individualised examination for candidates from the various medical schools recognised by the state one in materia medica and therapeutics only; (2) by adding an examination for all candidates in "internal medicine" exclusive of treatment, and with it associating "diagnosis;" (3) by making "pathology and bacteriology" a subject for examination by itself.

In addition it is suggested that the change of the term "the practice of medicine" to the "practice of the healing art" would enlarge the definition of the original term sufficiently to make it include all those who accept financial support from sick people, and would of necessity put all such under this one law requiring evidence of fitness before a license to practise is granted—a law which in our opinion the people of this state will never repeal.

Dr. Heffron moved the adoption of the resolution.

Motion seconded.

The President.—The resolution includes several recommendations. Shall we take them up separately, or as a whole?

Dr. Heffron.—The simple point about that is to arrange the examinations scientifically which are at the present time required by the state. Those familiar with the requirements of the state know they are physiology and hygiene, chemistry, pathology and diagnosis, anatomy, surgery, obstetrics and therapeutics, practice and materia medica. In other words, there is no place allotted for an examination in internal medicine. It is possible for a man to become a physician, according to the laws of the State of New York, without having shown any proof that he knows the natural history of disease.

Dr. W. A. HOWE, Ontario.—It seems to me that as a society we ought to adopt it and send it to the Committee on Laws of the State Society. I think that is the only course for us, and when the State society takes up the legislation, they consider it, and if they think advisable they present it to our law makers. I would second the motion.

Dr. Heffron.—This is simply suggested to the legislative committee of our State Society, and a similar memorandum could be sent to the legislative committee of the Homeopathic Society and the Eclectic Society. My opinion is that if a memorandum to this effect should be sent to those other societies that they would all be united in the application for the examination as stated there.

The motion was put to a vote and carried.

Dr. A. L. Benedict.—I would follow out that suggestion of the introducer and move that a memorandum of this be sent to the secretary of the State Homeopathic Society and the State Eclectic Society, and to the Medical Association of the State of New York.

Motion seconded and carried.

The Committee on Credentials recommended that the following delegates be elected to permanent membership in the association. Carried.

E. E. McKnight, Oswego; John C. Fisher, Chemung; J. P. Horley, Cayuga; W. T. Lum, Madison; C. M. VanDyke, Yates; S. R. Wheeler, Wm. B. Clapper, Ontario; I. N. Slingerland, C. S. Roberts, Wm. F. Law, A. S. Hotaling, W. L. Wallace, Dwight H. Murray, A. E. Larkin, G. J. Bryan, Onondaga; J. T. Sherman, L. E. Slayton, Wm. M. Barron, F. B. Andrews, M. J. Whiteside, Monroe.

C. E. Congdon, A. J. Colton, Erie.

The reading of papers was resumed and the following speakers announced:

The therapeutic value of the *x*-ray, C. A. Greenleaf, Rochester.

Deafness from scarlet fever and other sources, S. F. Snow, Syracuse.

Once more: unrecognised glaucoma—hopeless blindness, Wheelock Rider, Rochester.

Causes of death from strangulated hernia, William B. Jones, Rochester.

A case of specific intracranial lesion, involving the chiasm, causing somnolence, mental dulness and total blindness—hemiopic recovery, with normal visual acuteness, B. C. Loveland and F. W. Marlow, Syracuse.

The mind from a medical point of view, M. A. Veeder, Lyons.

The following papers were read by title:

Disinfection, Geo. W. Goler, Rochester.

Modification of the operation for inguinal hernia, J. P. Creveling, Auburn.

The influence of diseased tonsils on the health and development of children, John O. Roe, Rochester.

Appendicitis, A. A. Young, Newark.

Poisonings from unexpected directions, Francis E. Fronczak, Buffalo.

Diagnosis of hip-joint injuries, J. H. Jewett, Canandaigua.

Treatment of typhoid fever, A. L. Hall, Fulton.

A vote of thanks was tendered the Buffalo Society of Natural Sciences for the use of the assembly room, and another to Dr. Knowlton and Mr. Steele, of Syracuse, for their kindness in demonstrating the cardiac circulation. After which the president declared the meeting adjourned, to meet at Syracuse, N. Y., the third Tuesday of October, 1902.

